# Diagnostic Radiology

**DOI:** 10.1080/13577140120097120

**Published:** 2001-11


### DIAGNOSTIC RADIOLOGY

### 078 Image Fusion with PET and MRI: A New Modality in Evaluation of Bone and Soft Tissue Sarcoma

Annika Eigtved[1], Helle W Hendel[1], Markus Nowak[1], Karl Erik Jensen[2], Soeren Daugaard[3], John Gregor Pedersen[4], Catherine Rechnitzer[5], Carsten Thomsen[2], Birthe H Pedersen[2], Anders Krarup-Hansen[5]
*[1]PET & Cyclotron Unit, Rigshospitalet, Copenhagen University Hospital, [2]Dept. of Radiology, Rigshospitalet, Copenhagen University Hospital, [3]Dept. of Pathology, Rigshospitalet, Copenhagen University Hospital, [4]Dept. of Orthopedic Surgery, Rigshospitalet, Copenhagen University Hospital, [5]Dept. of Oncology, Rigshospitalet, Copenhagen University Hospital*

*Objective:* Characterization of sarcomas by FDG-PET can provide complementary information to MRI and CT. This may prove useful in more accurate evaluation of the tumor. Due to the heterogeneity of sarcomas, image fusion with PET and MRI can be optimal for biopsy-guidance. Furthermore, image fusion with PET and MRI can provide detailed anatomical localization of the functional changes in the tumor.
*Methods:* Ten patients with primary sarcomas have until now been included. Two were examined twice, before and after chemotherapy. FDG-PET scanning and MRI were performed with the relevant extremity fixed in a mould to ensure identical anatomy without deformation of the surface. A surface-based fitting algorithm was used for co-registering the PET transmission scans to the MRI data. The attenuation corrected emission scan was subsequently projected on the MRI scan.
*Results:* It was technically possible to co-register 9/12 PET and MRI examinations. Identical parameters as slice thickness, slice positioning and angulation are crucial. Two tumors expressed homogeneous activity in PET and homogenous signal intensity on MRI including a post-contrast study. The other seven were heterogeneous in both modalities. In four of these, MRI indicated a larger degree of tissue involvement than suggested by PET. In one, MRI indicated less malignancy than did PET. The results from the co-registration images were compared with histology and we found agreement in all cases between the grade of malignancy and the degree of FDG uptake.
*Conclusion:* In 4/9 examinations, MRI indicted a larger degree of tissue involvement than suggested by PET. These differences could not be detected without image fusion. Our preliminary results indicate, that image fusion with PET and MRI is feasible and has potential advantages compared to conventional MRI and FDG-PET alone in evaluation of sarcomas.

DOI: 10.1080/13577140120097120

